# Structural and rheological properties conferring fertilization competence to *Xenopus* egg-coating envelope

**DOI:** 10.1038/s41598-017-06093-3

**Published:** 2017-07-18

**Authors:** Mayu Hanaue, Naofumi Miwa

**Affiliations:** Department of Physiology, School of Medicine, Toho University, Ohmori-nishi 5-21-16, Ohta-ku, Tokyo 143-8540 Japan

## Abstract

The extracellular egg-coating envelope that comprises a meshwork of filaments polymerized by glycoproteins plays a pivotal role in species-selective sperm recognition and subsequent fertilization; however, the structural and rheological properties conferring fertilization competence to the egg-coating envelope remain poorly unveiled. Here we show several nanoscale-structural and viscoelastic properties of the egg-coat using the transmission electron microscopy and the quartz crystal microbalance experiments, following clamp of the egg-coat at either fertilization-competent or -incompetent statuses by short-term pretreatment with synthetic peptides. Individual filament of approximately 4.8 nm diameter crossed one another, forming several types of intersections. Higher competence-inducing treatment changed the proportion of V-, Y-, and T-type intersections, and induced more randomly deflected angles at intersections. Incompetence-inducing treatment increased the median of a Gaussian distribution of filament lengths that had a peak of 10–20 nm under control conditions; furthermore, this treatment created bumps in the 30–40 and 50–60 nm windows. Quartz crystal microbalance study revealed that viscoelasticity of the competent VE suspension was lower than that of incompetent VE, indicating that viscoelastic property required for successful fertilization resides within a specific range. These findings indicated that the architecture of the egg-coat is capable of rapid and dynamic remodeling, which determines fertilization efficiency.

## Introduction

Extracellular matrix (ECM) organizes the cellular microenvironment, and regulates cellular and/or cell-matrix interactions, thereby playing crucial roles in cell proliferation, differentiation, and migration in a variety of tissues, including skin, tendons, and blood vessels^1^. ECM constantly undergoes meshwork remodeling that influences cell-cell interaction and intracellular signaling, regulating cell behavior to maintain tissue homeostasis and sometimes to cause a local pathological microenvironments such as the cancer niche^2, 3^. In the reproductive system, the egg-coating envelope plays important roles in species-selective sperm recognition, fertilization and fertilized egg implantation. This egg-coating envelope, called the zona pellucida (ZP) or vitelline envelope (VE) in amphibians, contains a three-dimensional meshwork of filaments formed by polymerization of ZP proteins that are mainly secreted from growing oocytes with posttranslational modification by glycosylation^4, 5^. The egg-coat ZP protein isoforms include ZP1-4 in humans, ZP1-3 in mice, and gp120, gp69/64, gp41, and gp37in *Xenopus laevis* ^6^. These proteins associate with different ZP proteins via a conserved motif called the ZP domain, creating μm-long filaments that intersect, thereby forming a three-dimensional meshwork^7^. We recently found that dicalcin, a novel ZP protein-associated protein present in the intact VE of unfertilized eggs, suppresses the efficiency of fertilization through its interaction with gp41, a frog counterpart of mammalian ZP3^8^. The interacting regions between dicalcin and gp41 consist of two regions within dicalcin (nine and five amino acids in length; referred to as dcp11 and dcp15^9^) and a twenty-three amino acid region within gp41 (referred to as gpp2^9^). Synthetic peptides that corresponded to these regions dramatically affect fertilization by mimicking the action of each protein: short-term (~15 min) pretreatment with gpp2 neutralizes the action of native dicalcin, thereby increasing the fertilization rate, while treatment with two dicalcin-derived peptides (dcp11 or dcp15) exaggerates the action of dicalcin, thus decreasing this rate. Consequently, we considered that direct interaction between dicalcin and gp41 tunes the structural architecture of the VE meshwork by changing the binding affinity between ZP protein molecules, including gp41, gp37, and gp120, all of which constitute the VE meshwork. Indeed, pretreatment with these synthetic peptides altered *in vivo* lectin-staining patterns of the unfertilized egg-coating envelope for these two statuses (fertilization-competent and -incompetent), as well as the alignment pattern of the VE meshwork at electron microscopic levels; both results implied a functional role of dicalcin and gp41, as mentioned above^9^. Despite this evidence, however, it remains to be determined how structural properties of the VE, such as filament lengths and density of intersections, differ between high and low fertilization competence statuses, and how these changes in structural properties arise from filament remodeling. Nanoscale structural properties of filament meshwork have often been demonstrated for intracellular actin filaments, and these properties are known to reflect the structural basis for actin filament-related biological processes, including deformation, migration and locomotion^10, 11^. Contrary to these analyses of intracellular matrix (ICM) filaments, there has been no study characterizing structural properties of ECM filaments and correlating these properties with their biological function. Furthermore, a potential linkage between the egg-coat structural properties and its biophysical (*e.g*., rheological) properties remains unknown. Therefore, the objectives of this study were to investigate the nanoscale structural and microrheological properties of the fertilization competence of the ECM egg-coating envelope by using dicalcin- or gp41-derived peptides, and to determine the fine architecture of the egg-coating envelope and its viscoelasticity, both of which underlie fertilization competence, and these were accomplished by quantitative morphometric analyses using electron microscopy, and microrheological study using the quartz crystal microbalance (QCM) analysis.

**Figure 1:**
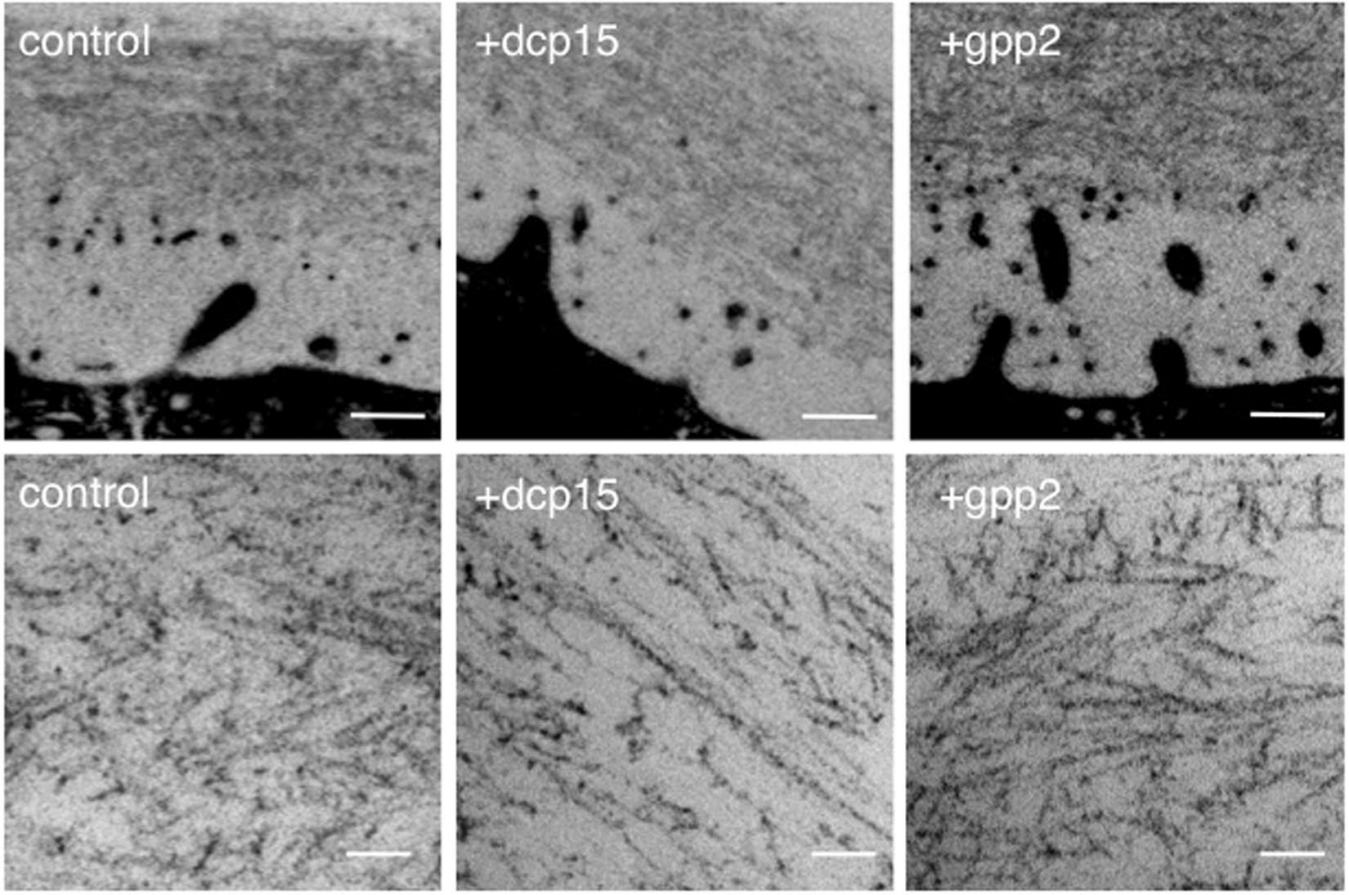
VE filament meshworks under different fertilization competent statuses. (Upper) Lower magnified TEM images treated with peptides (dcp1 as a control, dcp15 and gpp2; 4 μM; n = 3). Scale bar: 500 nm. (Lower) Higher magnified images. Scale bar: 60 nm.

**Figure 2:**
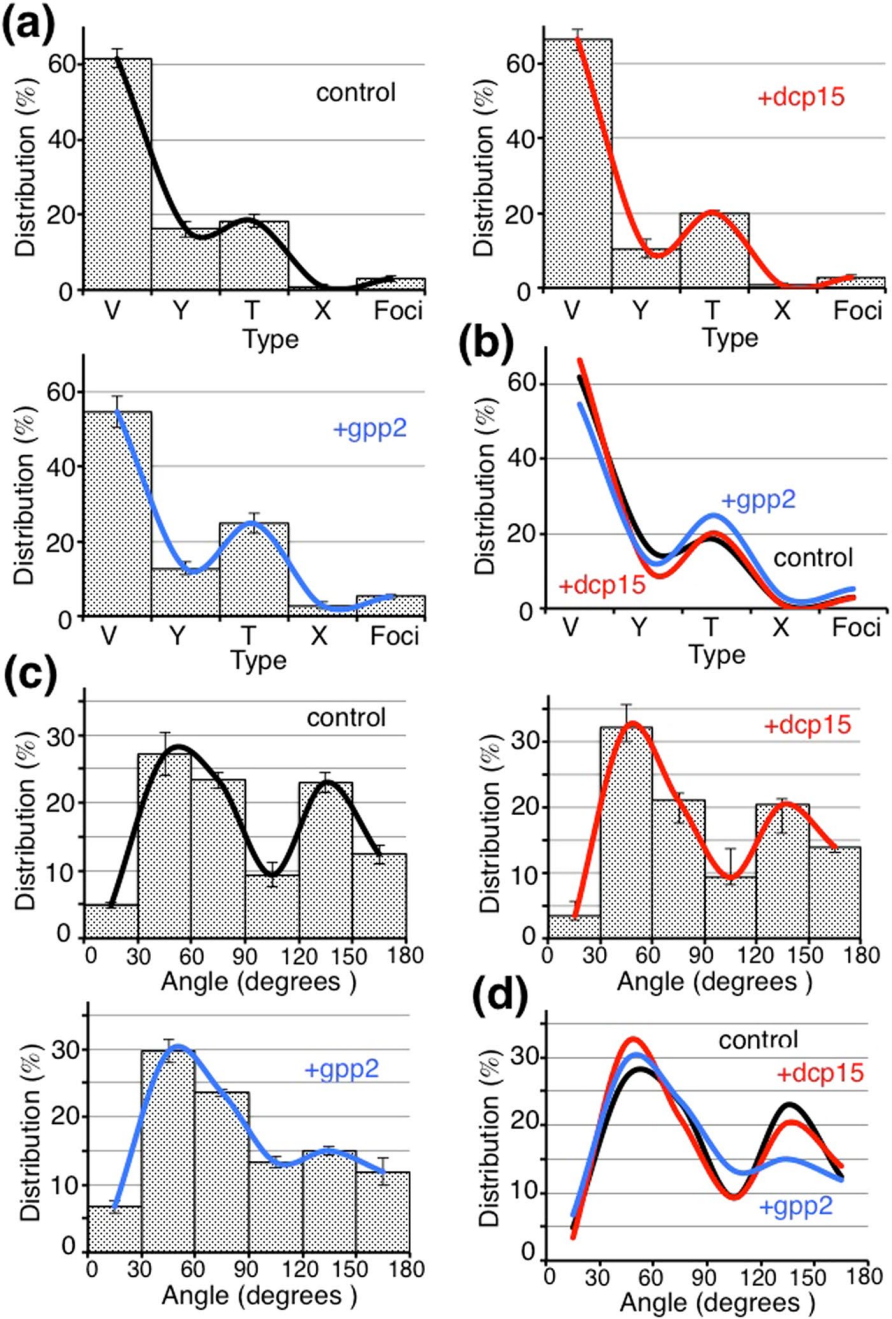
Distribution of intersection types and deflected angles. (**a**) Distribution ratio of intersection types, including V-, Y-, T-, X- and Foci types (control, n = 317; +dcp15, VE pretreated with dcp15, n = 224; +gpp2, VE pretreated with gpp2, n = 435). Averaged data were also fitted (mean ± SEM, n = 4). (**b**) Fitted curves were overlaid (Black, Red and Blue for control, dcp15 and gpp2, respectively). (**c**) Deflected angles at intersection were measured (control, n = 365; +dcp15, VE pretreated with dcp15, n = 318; +gpp2, VE pretreated with gpp2, n = 677). Averaged data were also fitted (mean ± SEM, n = 4). (**d**) Fitted curves were overlaid (Black, Red and Blue for control, dcp15 and gpp2, respectively).

**Figure 3:**
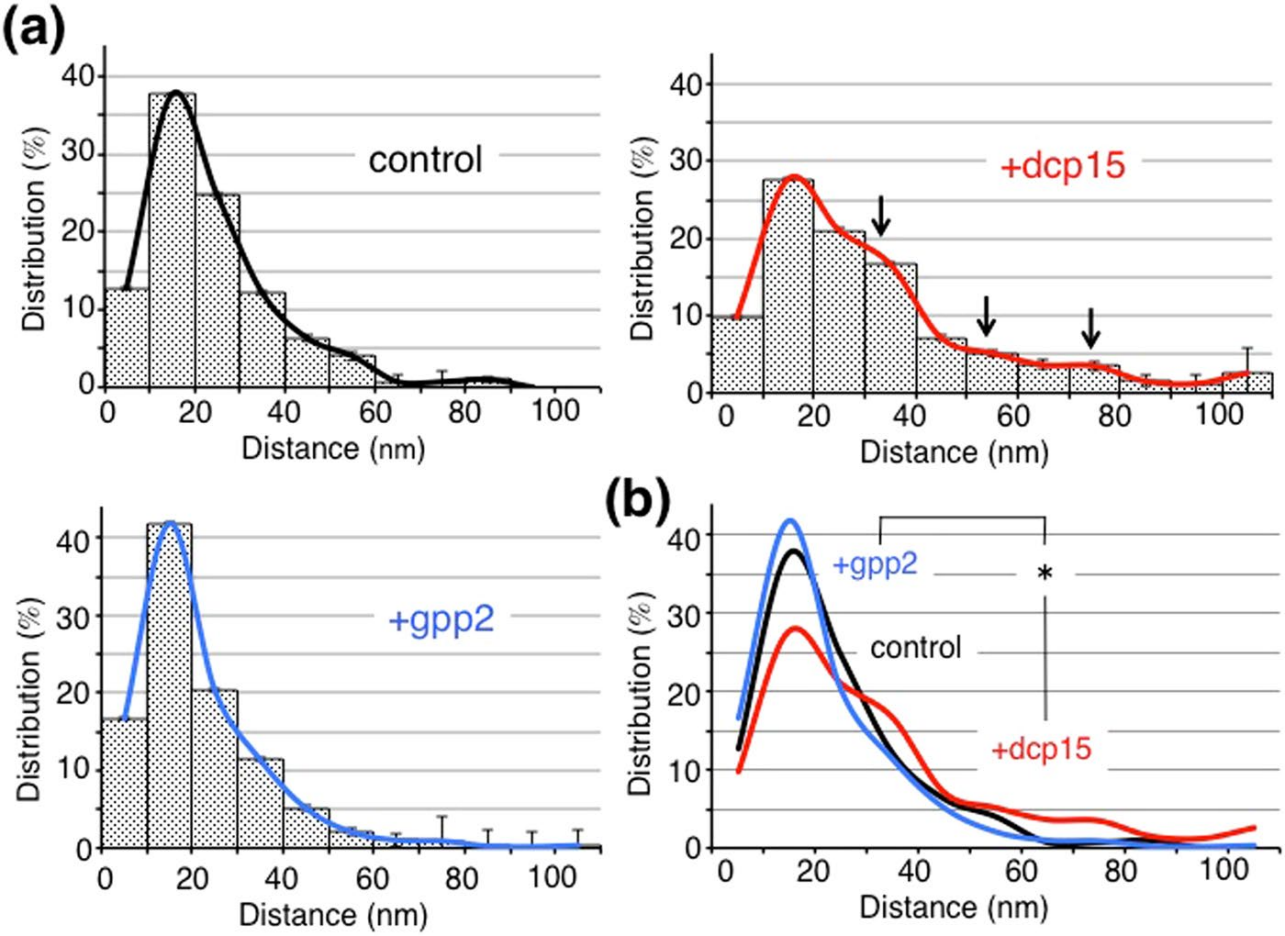
Distributions of lengths between intersections. Histograms of lengths between intersections were plotted (control, +dcp15, +gpp2), and averaged data were also fitted (mean ± SEM, n = 4). The histogram for dicalcin-derived peptide-treated VE exhibited bumps at windows of 30–40, 50–60, and 70–80 nm (arrows). Fitted curves were overlaid (Black, Red and Blue for control, dcp15 and gpp2, respectively).

We first characterized the general properties of the *Xenopus* VE filament meshwork. VE filaments were homogeneously distributed within the VE (Fig. 1, left). Most of the VE filaments appeared straight between intersections, and deflection of the filament sometimes occurred at an intersection, causing the filament to adopt a zigzag shape. Consistent with our previous report^9^, VE filaments pretreated either with dcp11 or dcp15 (incompetence-inducing peptides) were aligned in parallel to the egg plasma membrane, giving the appearance of a “pin-stripe” pattern (Fig. 1, middle). In contrast, VE filaments pretreated with gpp2 (high competence-inducing peptide) were randomly aligned at oblique angles to the egg membrane (Fig. 1, right). In every VE status (control, incompetent, and highly competent VEs), we found five types of filament intersection, namely V-, Y-, T-, X- and foci-type (Supplementary Fig. S1a). For the control VE group, ~61%, ~16% and ~18% of all intersections were V-, Y- and T-types, respectively (n = 317). The V-, Y-, and T-types constituted ~95% of all intersections; the X- and foci-types represented only ~5% of intersections (Fig. 2a). Note that we considered V-, Y- and T-type intersections in our two dimensional TEM images as “real” intersections, and performed the quantitative analyses using these three types (for details, see Materials and Methods). At intersections, filaments deflected, and their angles varied with two preferences of 30–60° and 120–150° in all three conditions (Fig. 2c, n = 365). Lengths between intersections exhibited a Gaussian distribution, with a peak distribution at 10–20 μm, and exhibited gradual attenuation at larger distances (Fig. 3, control, n = 723). The average diameter of each filament was ~4.8 nm, exhibiting no significant difference between treatments, suggesting that peptide treatment did not significantly change the molecular components of an individual filament (*i.e*., the molar amount of ZP proteins that constitutes a unitary length of filament) (Supplementary Fig. S1b).

To investigate structural differences among three VE statuses (control [cVE], highly competent VE [hVE], and incompetent VE [iVE]), we determined distribution ratios of intersection types, following clamp of the egg-coat by short-term pretreatment with synthetic peptides. Dcp15-treated VE (iVE) exhibited a distribution of ~66% V, ~10% Y, and ~20% T-type intersections (n = 224), which was similar to that of control (Fig. 2a, control). Gpp2-treated VE (hVE) showed a significantly different distribution of ~54% V, ~13% Y, and ~25% T-type intersections (Fig. 2a, +gpp2; n = 435). Gpp2 treatment significantly decreased the proportion of V-type (~54%) and increased the proportion of T-type (~25%) intersection, compared with the other two conditions. This observation indicates that a rapid transition from V-type to T-type intersections occurred in response to brief treatment (~15 min) with gpp2, implicating generation of a new branch at intersections. Next, we evaluated the angles of intersecting filaments. Deflection angles between crossed filaments varied with two preferences of 30–60° and 120–150° in all three conditions (Fig. 2c). Since maximal angles at T-type would be 90° (see Supplementary Fig. S1a), measured angles over 90° mostly belonged to V- and Y-type intersections. Interestingly, the proportion of 120–150° angles decreased significantly in gpp2-treated VE, compared to the control (15% and 22% for hVE and cVE, respectively, Fig. 2d), whereas the proportion of 90–120° angles increased (12% and 9% for hVE and cVE, respectively). Moreover, we investigated the branch angles at every type of intersection, and found an evenly distributed pattern in V- and T-type intersections (Supplementary Fig. S2). These results indicated a more even distribution of branch angles in response to gpp2 treatment, which suggested that braches at intersections of hVE are formed more randomly or arbitrarily, and they may have a greater flexibility than those of iVE. Accordingly, these results emphasized the fact that rapid and dynamic remodeling of VE filament meshwork is caused by manipulation of the action of dicalcin and gp41 (for a scheme, see Supplementary Fig. S3).

We next measured distances between V-, Y- and T-type intersections, and found that the distance distribution for dcp15-treated VE (iVE) was significantly different from the distributions for the other two conditions (Fig. 3) (p = 0.008, χ^2^ test, n = 4). Control VE (cVE) and gpp2-treated VE (hVE) exhibited median values of ~20 nm (n = 723 and 1040, respectively), while dcp15-treated VE (iVE) had a median of ~28 nm (n = 836). This observation indicated that distances between intersections for iVE tend to be longer than those for the other two conditions. Noteworthy is that, besides the largest peak at 10–20 nm, there were bumps in the windows of 30–40 nm and 50–60 nm for the iVE, suggesting that a 10–20-nm-length filament constitutes the unitary length for VE remodeling, and this unitary “module” would connect with another filament, enabling stepwise filament elongation (*i.e*., twice or three times length of the unitary “module”). Extrinsically-added dicalcin binds to gp41, and dicalcin molecules at the ends of two filaments may associate with each other, mediating inter-filament connection. Otherwise, dicalcin binds to ZP proteins, enhances aggregation and concentration of filaments to induce partial association, ultimately resulting in reorganization of the VE meshwork (for a scheme see Supplementary Fig. S3a,b).

**Figure 4:**
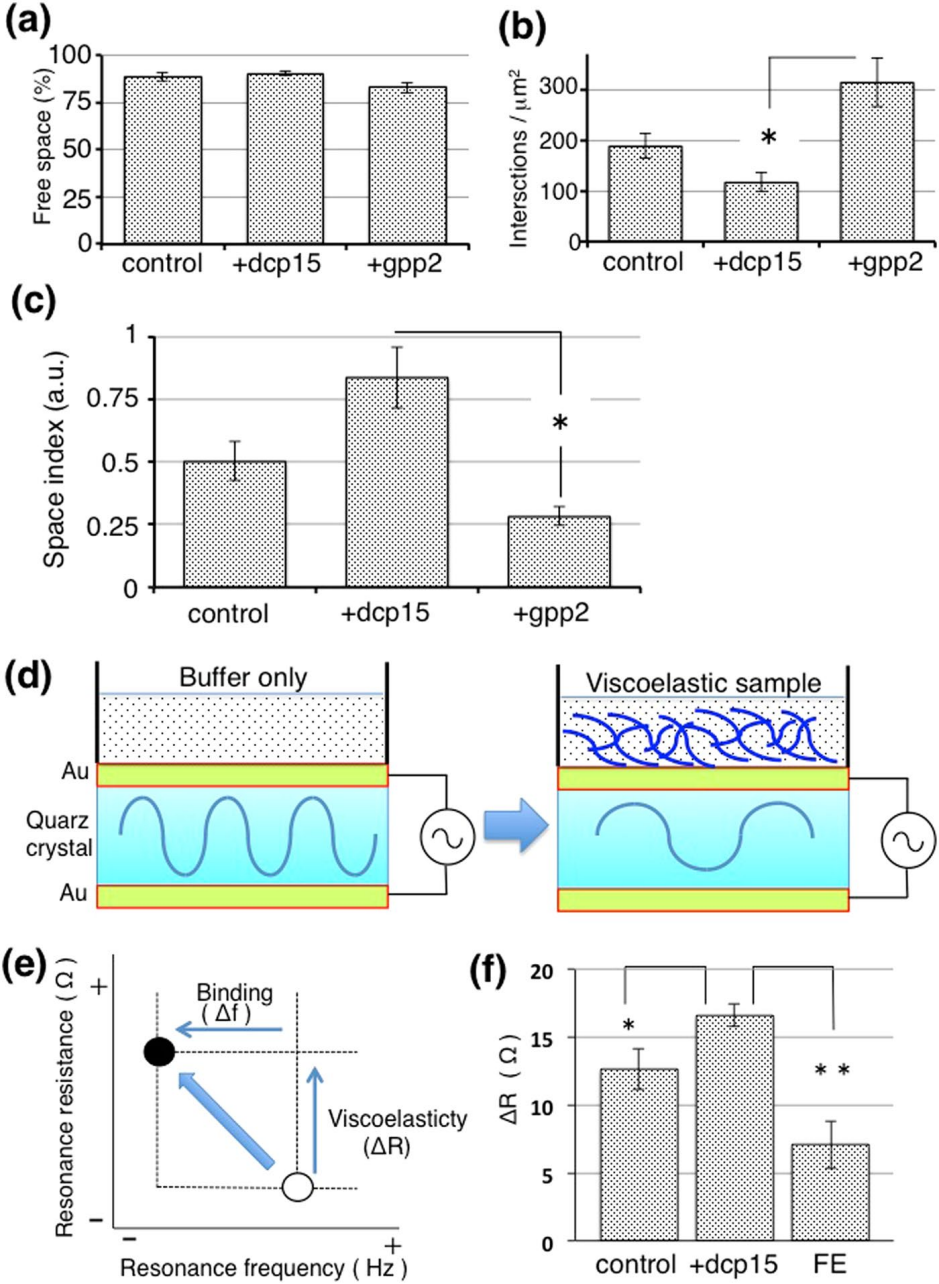
Filament-free area and VE suspension viscoelasticity correlate with fertilization competence. (**a**) To examine the correlation of filament-free area with fertilization competence, we first measured the ratio of filament-free area against the total area (mean ± SEM, n = 4). (**b**) The number of intersections per area was measured (mean ± SEM; *P = 0.008, Student’s *t*-test, n = 4). (**c**) The ratio of free-space/number of intersection (mean ± SEM; *P = 0.008, Student’s *t*-test, n = 4). Filament-surrounded free space of fertilization competent VE is significantly smaller than that of fertilization incompetent VE (mean ± SEM; *p = 0.04, Student’s *t*-test, n = 30), suggesting that fertilization competence is correlated with the filament-surrounded free space. (**d**) A scheme that represents QCM analysis. Alternating voltage induces the oscillation of the quartz crystal. When a supension of viscous VE is placed on the surface of the quartz crystal, the resonance properties of the quartz oscillation change. (**e**) A scheme that represents changes in parameters at QCM analysis. Viscous sample evokes an increase in resonance resistance (dR) and a decrease in frequency (df), each of which reflects the viscoelasticity, and the binding to the quartz surface, respectively. (**f**) Fertilization competence-dependent changes in resonance resistances. Increases in resonance resistance were averaged (mean ± SEM; *P = 0.03, **P = 0.005, Student’s *t*-test, n = 6–10). Dcp15-treated incompetent VE showed a greater resistance (*i.e*., greater viscoelasticity) than the control VE, indicating that dicalcin increased the viscoelasticity of the VE. In addition, the FE showed a lower resistance than control, indicating that FE has lower viscoelasticity. Thus, viscoelasticity for successful fertilization resides within a limited range between the iVE and FE values (*i.e*., 7.5–16 Ω).

Our previous study suggested that dicalcin preferentially affected the sperm penetration process rather than the initial sperm-ZP binding^8^, and accordingly the architecture of the dcp15-treated VE (*i.e*., iVE under the excess degree of dicalcin’s action) would hamper the acrosome reaction (AR) with high probability^9^. Although there is no evidence of a direct linkage between VE structural architecture and sperm AR, the potential effect(s) of physical size of moving trait on sperm AR has been reported in a study, showing increased AR occurrence *in vitro* when sperm penetrated the polycarbonate filter having a 3-μm pore size^12^. Therefore, the volume of space surrounded by individual VE filaments could be relevant to AR induction. To investigate the filament-surrounded area in fertilization-competent or incompetent statuses, we first measured the filament-free area (Fig. 4a), and next examined the number of intersections per observed area (μm^2^) as an intersection density (Fig. 4b). By using these two parameters, we calculated the arbitrary space index (i.e., space area/number of intersections) (Fig. 4c), and found that the filament-surrounded free space within hVE (+gpp2) was significantly narrower than that of iVE (+dcp15) (0.28 and 0.83 for hVE and iVE, respectively; n = 30, p = 0.024, Student’s t-test), which suggested that fertilization competence is correlated with the filament-surrounded free space, and the narrower space would preferentially favor the occurrence of sperm AR.

We then questioned whether these structural properties influence biophysical property of the VE, and examined the viscoelasticity of the VE suspensions by microrheological study using the quartz crystal microbalance (QCM) analysis (Fig. 4d,e). This analysis measured apparent resonance resistance of small volumes (~180 μL) of the specimen via quartz surface oscillation, and a change in resonance resistance is known to be proportional to its viscoelasticity^13, 14^. Dcp15-treated VE (iVE) showed a greater resistance (*i.e*., greater viscoelasticity) than the control VE in which dicalcin was absent due to repeated rinse during VE preparation (~12 Ω and ~16 Ω, for control and iVE; *p = 0.01, n = 9) (Fig. 4f), indicating that dicalcin increased the viscoelasticity of the VE. In addition, we examined the viscoelasticity of the egg-coating envelope following fertilization (termed the fertilization envelope: FE). After the cortical granule exocytosis triggered by fusion of spermatozoon and egg, ZP proteins undergo modification, possibly limited proteolysis by the exudates from the cortical granule, and the VE ultimately converts to the sperm-impenetrable FE, a blockade against polyspermy^15, 16^. We found that the FE showed a lower resistance (~7.5 Ω) than the control (Fig. 4f), indicating that FE has lower viscoelasticity. Thus, viscoelasticity for successful fertilization resides within a limited range between the iVE and FE values (*i.e*., 7.5–16 Ω in *X. laevis*). It remains to be determined whether this range (equivalent to ~12–15% of glycerol viscoelasticity; data not shown) is similar or dissimilar among species. If dissimilar, it would be interesting to examine whether the viscoelastic range could be a biophysical discriminatory zone that acts as a species-selective functional “filter” between sperm and the egg coat.

The viscoelasticity of the ICM filament meshwork has been characterized for actin filaments, and it rapidly changes in parallel with cellular activities, even during short-term events such as migration and locomotion (*e.g*., second level or less)^17, 18^. Viscoelasticity is also known to correlate with histochemical parameters at electron microscopic levels (*e.g*., intersection density, angles and free space), as consequences of ICM remodeling^10^. Filament-associated proteins (*e.g*., actin-binding protein for actin filament) have been known to modify these parameters by initiating or terminating filament growth, affecting polymerization rates, crosslinking filaments or dividing into smaller units^19, 20^. Analogous to the case of the actin filament, our present study of the ECM egg-coating envelope found that dicalcin, a VE meshwork-associated protein, regulates the three-dimensional structure of the VE meshwork; this protein also influences histochemical properties (*e.g*., intersection density, angles and free space) and viscoelasticity, ultimately affecting the fertilization efficiency. These results are the first to demonstrate a remarkable characteristic of the ECM egg-coating envelope: this ECM has a capacity of undergoing a dynamic and rapid remodeling by extrinsic treatment. Furthermore, our demonstration is applicable not only for development of efficacious contraceptive drugs and treatments toward animal infertility, but for gaining a novel insight into the mechanism of ECM-cell interaction and novel therapeutic approaches through extrinsic control of the ECM architecture.

## Materials and Methods

### Animals

All animal experiments were approved and in accordance with the animal care committee’s at Toho University.

### Transmission electron microscopic analyses

Dejellied *Xenopus* eggs were pre-incubated with either of dicalcin-derived (dcp1 as a control, dcp15) or gp41-derived (gpp2) peptides at a concentration of 4 μM for 15 min, and fixed in 2% glutaraldehyde overnight at 4 °C. Fixed eggs were refixed with 2% OsO_4_ and dehydrated with a graded series (30–100%) of ethanol. Gelatine capsules containing epoxy resin (Quetol 812, Nisshin-EM, Tokyo, Japan) were inverted onto the specimens. After the resin was cured at 60 °C for 48 h, the capsules were detached. Ultrathin sections (~60 nm thickness) were stained with uranyl acetate and lead acetate, and observed using a Hitachi H-7600 (Hitachi, Tokyo, Japan) at 100 kV.

### Two-dimensional morphometry

VE filaments were found to distribute evenly in the entire ZP field, and often overlapped one another. In the present study, we observed diameters of each filaments, shapes of intersections, intersection angles, and inter-intersection lengths, filament-free space. Several types of intersections were observed, and we classified them into the following four types; V type (two filaments stopped at the identical position), Y type (three filaments ended on one junction), and T type (two filaments crossed, but ended on the other filament), X type (two filaments crossed over), and Foci (filament junction that involve more than three filaments) (for a detailed description of each types, see Supplementary Fig. 1). Since each filaments has a diameter of ~4.8 nm (Supplementary Fig. 2), and prepared sections have thicknesses of ~40-~60 nm, we considered that observed filaments may be in a skew position, thereby causing a “false” cross. This situation may occur for X and Foci types. However, most (~95%) of the intersections were V-, Y- and T-types, and for these three types, one filament rarely ended at the exactly identical location of other crosses. We, therefore, focused on V-, Y-, and T-intersections when evaluating filament properties. Free filament ends are rare, and these filament ends are reflecting to project orthogonally upward from the body of the network or extend down to the substrate; therefore, this study did not evaluated them. To estimate filament-free area in our TEM images, we picked the intensity value at the center of arbitrary filament-free areas, and measured the area for which the intensity was below this level in the TEM image (size, 400 nm × 400 nm; intensity, 8 bit). Note that this measurement may underestimate the area of “free space” (*e.g*., the actual free space may be larger than our estimation). All measurement were performed blind to experimental conditions.

### Preparation of the egg-coating envelope

VE proteins of *Xenopus* unfertilized eggs were prepared by sieving method described elsewhere^21, 22^. Briefly, envelopes were collected by passing dejellied egg-lysate through a 100-μm filter and the filter was washed extensively with distilled water. Isolated envelopes were stored overnight in 2 M NaCl, 2 mM CaCl_2_, 10 mM Tris-HCl (pH 7.4) to solubilize contaminants^23^ and heated at 70 °C before use. FE proteins were prepared similarly with the above, following egg activation by addition of ionophore (A23817, 5 μM) as described elsewhere^15^.

### Quartz crystal microbalance analysis of the suspension of the egg-coating envelope

To measure rheological properties of viscous VE or FE suspensions, we performed quartz crystal microbalance (QCM) analysis using 9 MHz AT-cut quartz crystals (5-mm diameter, 150-nm thickness) (QCM922A, SEIKO EG & G, Tokyo, Japan). A 180-μm suspension of VE or FE proteins (gp41 concentration: 5 μM) was placed onto the quartz surface after blocking non-specific binding by BSA at room temperature, and resonance resistance was measured. Resonance resistance of buffer sample (10 mM Tris pH7.4, 100 mM NaCl) was set to be 0. We also performed QCM analysis using aqueous glycerol solutions as viscosity standards, and estimated equivalent glycerol concentrations to QCM values of VE or FE suspensions.

## Electronic supplementary material

Supplementary Information
