# Supplementary material for: Structural and rheological properties conferring fertilization competence to Xenopus egg-coating envelope
Source: Sci Rep. 2017 Jul 18;7:5651. doi: 10.1038/s41598-017-06093-3 (PMC5515883; doi:10.1038/s41598-017-06093-3)
Supplement: Supplementary file 1 — Supplementary Information [file 41598_2017_6093_MOESM1_ESM.pdf]

Supplementary Information

Structural and rheological properties conferring fertilization competence to *Xenopus* egg-coating envelope

Mayu Hanaue, Naofumi Miwa

Department of Physiology, School of Medicine, Toho University, Ohmori-nishi 5-21-16, Ohta-ku, Tokyo 143-8540, Japan.

Supplementary Fig. S1 Types of intersections and comparison of filament diameter

(**a**) Intersections are classified into four types, including V-, Y-, T-, X- and Foci-type. Representative photos for each types are shown. Angles at intersections were measured as indicated, and for V- and T-types, smaller angles were taken for our study. (**b**) The average diameter of each filament was ~4.8 nm, showing no significant difference between control and peptide-pretreated VEs, indicating that neither of the peptides pretreatments alter the molar amount of ZP proteins that constitutes a filament per length, and therefore, neither of the peptide pretreatments are unlikely to attach or detach de novo ZP proteins to or from an individual VE filament.

Supplementary Fig. S2 Distribution of deflection angles at every type of intersections in dcp15- and gpp2-treated VEs

Distribution ratio of deflection angles at V-, T-, and Y-type intersections in the

dcp15-treated VE (+dcp15) and gpp2-treated VE (+gpp2). Averaged data were also fitted (mean±SEM, n=3). (**d**) Fitted curves were overlaid (Red, +dcp15; Blue, +gpp2). Deflection angles of intersections in dcp15-treated (i.e., incompetent) VE varied with some preferences (*e.g.*, 30-60° and 120-150° for V-type, 30-60° for T-type), whereas those in gpp2-treated (*i.e.*, highly competent) VE varied more evenly. These results suggested that branches at intersections of hVE are formed more randomly or arbitrarily, and they may have a greater flexibility than those of iVE.

Supplementary Fig. S3 Schemes for VE meshwork remodeling

(**a**) Change in the distance between intersections. The histogram of the distances between intersections exhibits bumps in every 10-20 nm, indicating that smaller filaments of 10-20 nm act as units to create longer filaments; therefore we considered a unitary branch that has already existed to be inserted and assembled into a longer filament as a duplet, triplet or multimeric structure. (**b**) We depict potential patterns of remodeling as follows; (i) V-Y type, a unitary V-type is inserted into Y-type, creating a double-length filament; (ii) Y-T type, Y-type is inserted into T-type, creating a double-length filament; (iii) V-Y type, a unitary V-type is inserted into Y-type that has a double-length branch, creating a triple-length filament.

Supplementary Fig. S4 Hypothesized model of the action of dicalcin

(**a**) Hypothesized model of the action of dicalcin to mediate the crosslink of VE filaments. VE filaments are composed of multimeric ZP proteins (green and yellow for

gp41 and gp69/64, respectively), and gp41 binds to dicalcin (pink) in the native VE. Dicalcin belongs to the S100 protein family, which is one of the major calcium-binding protein family in a variety of tissues[1,2]. Since some S100 proteins has been known to associate with each other to form multimers[3], dicalcin molecules at ends of the two filaments may associate with each other under certain VE conditions (*e.g.*, under the excess amount of dicalcin), mediating a connection between two VE filaments to generate longer-length filament.

(**b**) Besides the above scenario for direct linking by dicalcin, there may be another possibility in that dicalcin exerts an indirect effect on elongation of filament. Dicalcin binding to ZP proteins may enhance interaction between ZP proteins, and increase the concentration of the molecules to aggregate filaments, which ultimately leads to connection of the filaments.

1. Heizmann, C.W., Fritz, G. & Schäfer, BW. S100 proteins: structure, functions and pathology. *Front Biosci*. **7**, 1356-68 (2002).
2. Donato, R. Intracellular and extracellular roles of S100 proteins. *Microsc Res Tech*. **60**, 540-51 (2003).
3. Franz, C., Durussel, I., Cox, J.A., Schäfer, B.W. & Heizmann, C.W. Binding of Ca^{2+} and Zn^{2+} to human nuclear S100A2 and mutant proteins. *J Biol Chem* **273**, 18826-18834 (1998).

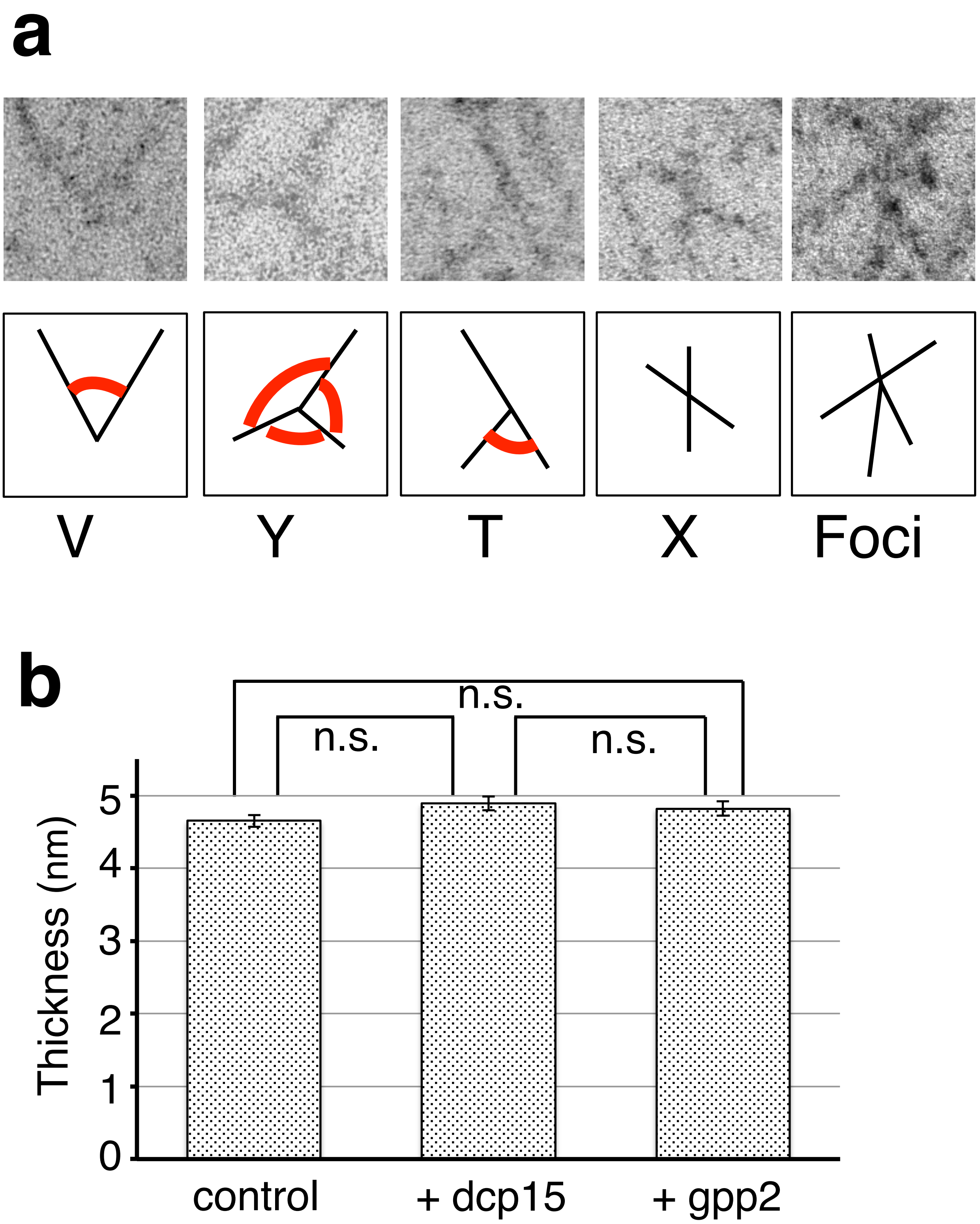

Supplementary Fig.S1 Hanaue et al.

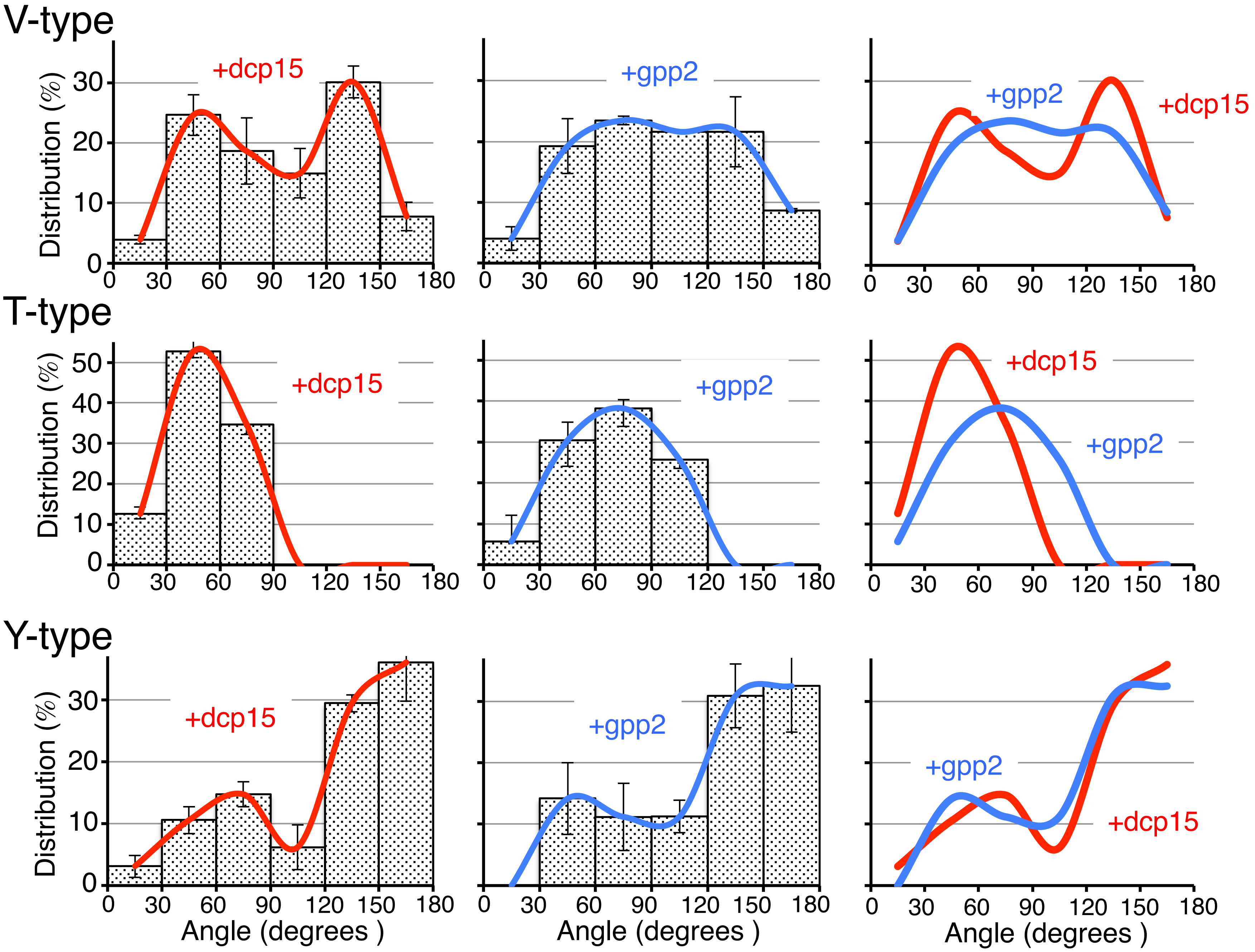

Supplementary Fig. S2 Hanaue et al.

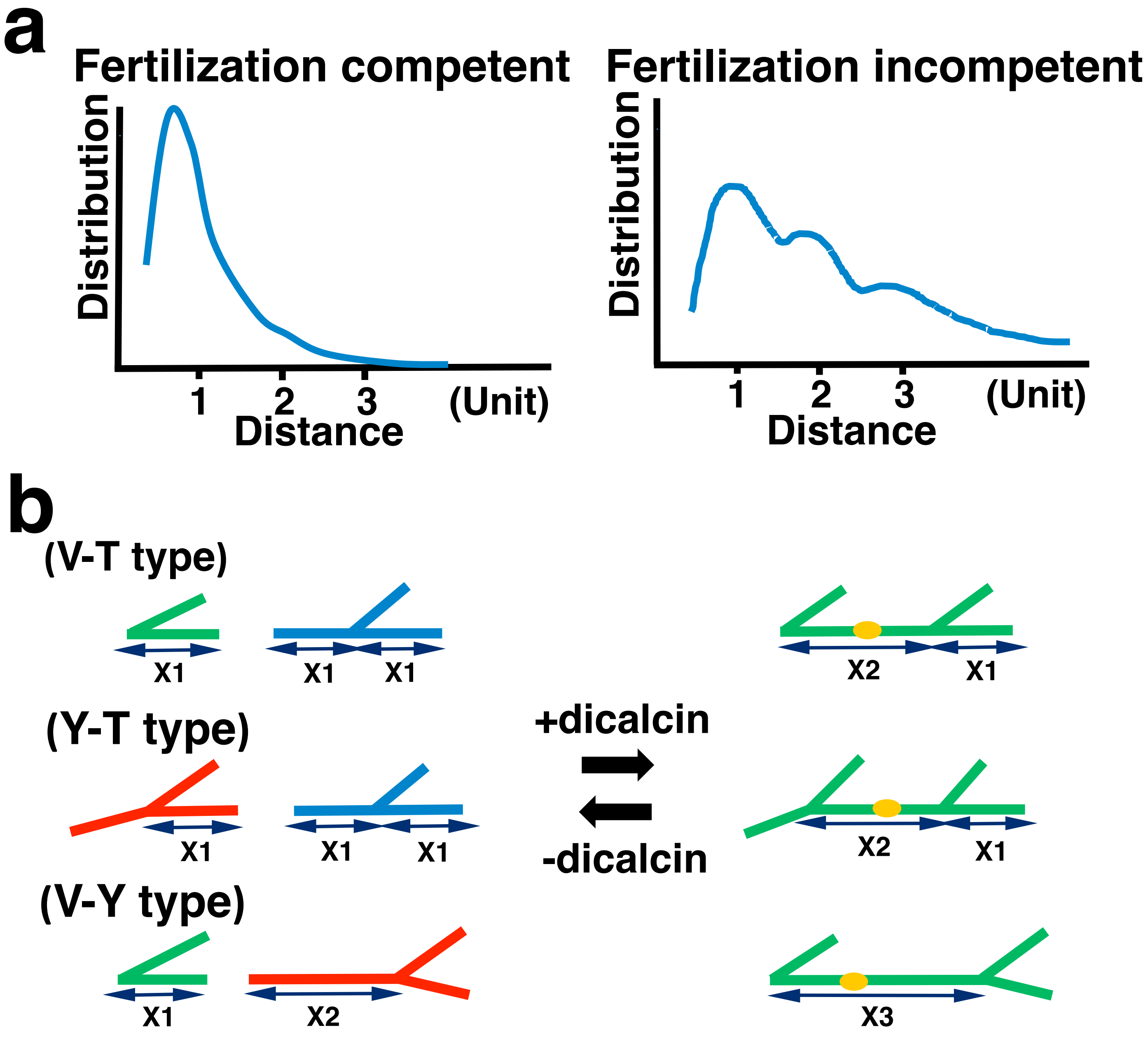

Supplementary Fig. S3 Hanaue et al.

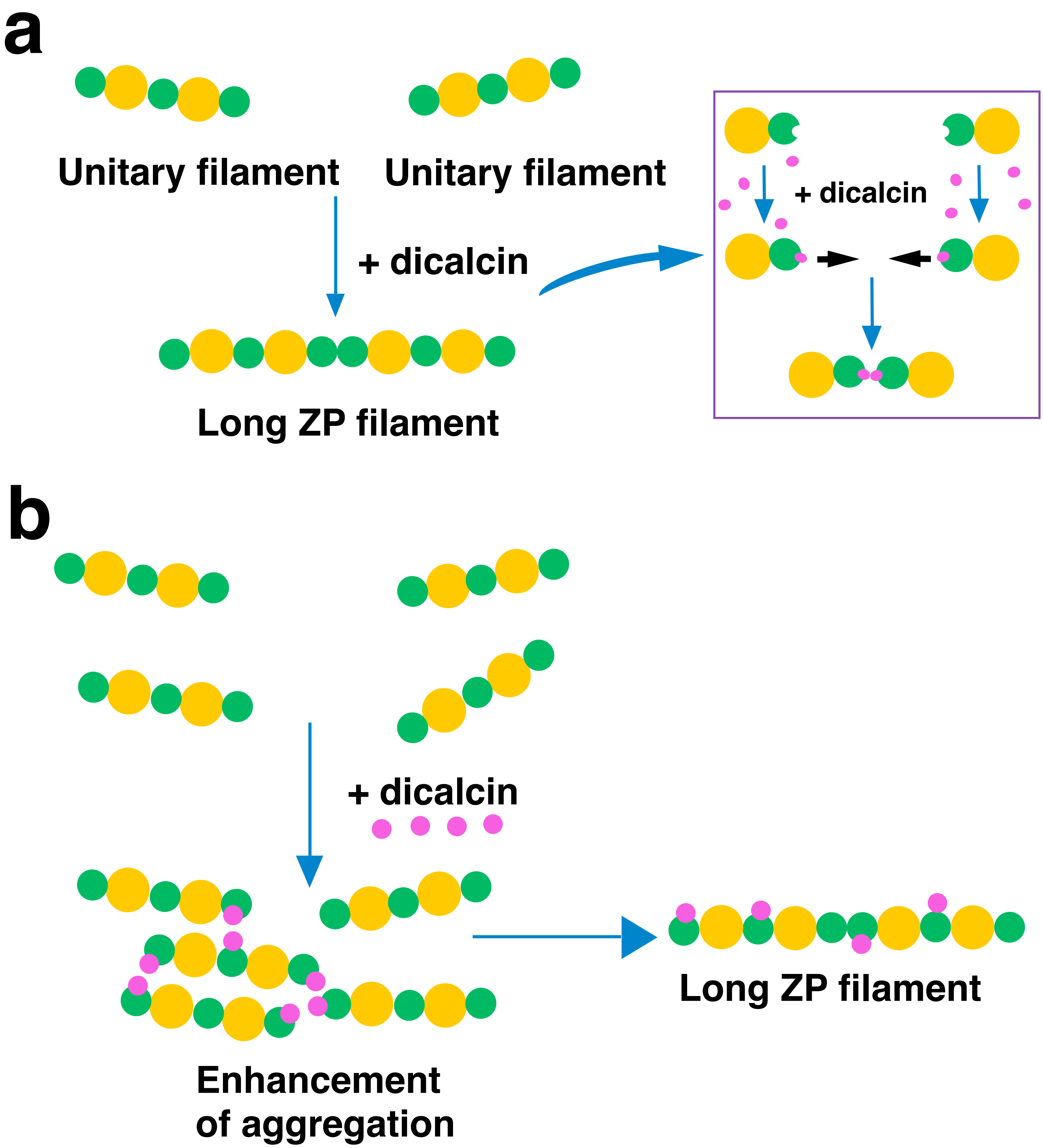

Supplementary Fig. S4 Hanaue et al.
